# Product presentation in the live-streaming context: The effect of consumer perceived product value and time pressure on consumer’s purchase intention

**DOI:** 10.3389/fpsyg.2023.1124675

**Published:** 2023-02-08

**Authors:** Nan Zhang

**Affiliations:** School of Economics and Management, Beijing Jiaotong University, Beijing, China

**Keywords:** product presentation, live streaming, perceived product value, time pressure, purchase intention

## Abstract

Live streaming is conducive to consumers obtaining rich and accurate product information, by displaying products through real-time video technology. Live streaming provides a new type of product presentation method, such as showing products from different perspectives, interacting with consumers by trying the products out, and answering consumers’ questions in real time. Other than the current research focus on anchors (or influencers) and consumers in live-streaming marketing, this article tried to explore the way of the product presentation and its effect and mechanism on consumers’ purchase intention. Three studies were conducted. Study 1 (*N* = 198, 38.4% male) used a survey to explore the main effect of product presentation on consumers’ purchase intention and the mediating effect of the perceived product value. Study 2 (*N* = 60, 48.3% male) was a survey-based behavioral experiment, and it tested the above effects in the scenario of food consumption. Study 3 (*N* = 118, 44.1% men) tried to deeply discuss the above relationship in the appeal consumption scenario by priming different levels of the product presentation and time pressure. The results found that the product presentation positively affected consumers’ purchase intention. The perceived product value played a mediating role in the relationship between product presentation and purchase intention. In addition, different levels of time pressure in the living room moderated the above mediation effect. When time pressure is high, the positive impact of product presentation on purchase intention is strengthened. This article enriched the theoretical research on product presentation by exploring product presentation in the context of live-streaming marketing. It explained how product presentation could improve consumers’ perceived product value and the boundary effect of time pressure on consumers’ purchase intention. In practice, this research guided brands and anchors on designing product displays to improve consumers’ purchase decisions.

## Introduction

When consumers shop online, they cannot directly view, touch, taste, or try products. Therefore, the product presentation information becomes the critical clue for consumers to judge the product quality and make purchase decisions (Jiang and Benbasat, 2004). The e-commerce platforms try to optimize product presentation to effectively convey related product information to consumers, such as using traditional text, pictures, animation, voice, background music, and video (Jovic et al., 2012). Distinct formats of product presentation provide different influences on consumers’ cognition, emotion, and behavior. It has been proved that the high media richness presentation could significantly reduce the perceived risk and improve consumer trust (Yue et al., 2017) and consumer product preference (Jovic et al., 2012).

With the rapid development of live-streaming marketing, the living room provides a new style of product presentation in real-time 3D formats. Extent research on product presentation mainly focused on designs on the webpage of e-commerce, namely, the 2D display and prerecorded video (Algharabat et al., 2017; Petit et al., 2019). However, few researchers have examined the effect and specific mechanism of the live-streaming product display formats on consumers’ decisions. Product presentation in live streaming is different from that on a traditional e-commerce webpage. The product presentation in live streaming provides rich visual information by displaying products from multiple angles and sensory information by trying the product. The interactive technology used in live streaming helps to increase consumer engagement, time sensitivity, and personalized shopping experience (Sjöblom and Hamari, 2017). In addition, real-time interactions, such as displaying products according to consumers’ requests and answering questions in a targeted manner, make consumers feel like shopping in physical stores (Kumar and Tan, 2015). Given the difference in product presentation between webpage and live streaming, it is necessary to explore how products should be presented in live streaming and the effect on consumer behavior.

There is also a research gap on the research objects of live streaming. Theoretically, live-streaming marketing mainly focused on the characteristics of anchors and the interaction between anchors and consumers on consumer decisions. The influencing factors include anchor type (Huang et al., 2021), fit between anchor and products/brand (Park and Lin, 2020), interactional communication style (the sense of community and emotional support; Chen and Liao, 2022; Liao et al., 2022), consumer’s social motivation (Hilvert-Bruce et al., 2018), and consumer’s state boredom (Zhang and Li, 2022). However, less research paid attention to the effect of products.

In addition, the mechanism of product presentation in live streaming on consumer’s decisions may change. Prior studies explained the specific mechanisms of product presentation on consumer’s purchase intention, such as mental imagery (Overmars and Poels, 2015; Flaviaìn et al., 2017), perceived diagnosticity (Cheng et al., 2022), and perceived risk (Fiore et al., 2005; Kim and Forsythe, 2008; Cano et al., 2017). Moreover, researchers also explored the moderating effect of both consumer factors and product factors, such as information processing motivation (Orús et al., 2017), need for touch (Flaviaìn et al., 2017), the product type (Li and Meshkova, 2013; Huang et al., 2017), and product rating (Cheng et al., 2022). However, the above findings might not explain the psychological mechanism of consumers’ decisions in the context of real-time 3D product presentation in the live room. Therefore, this article will test the mediating effect of consumer perceived product value and the moderating effect of time pressure.

Specifically, this research focused on product presentation in the context of live streaming, and it intended to address the following research questions. Could product presentation, rather than the prevalent influence of anchors, promote consumer decisions in the live room? How does the product presentation increase consumers’ purchase intention? Does time pressure strengthen or weaken the positive effect of product presentation on consumers’ purchase intention? Based on the theoretical analysis and practical observation, this research proposed that a high level of product presentation increases consumer purchase intention, through the mediating effect of consumer perceived product value. The boundary effect of the above relationship is the perceived time pressure in the live room.

## Theoretical background and research hypotheses

### Online product presentation

Different from shopping offline, shopping online could not provide an equivalent tactile experience in physical stores (Cano et al., 2017). According to information processing at the cognitive level, consumers need to acquire, process, retain, and retrieve information (Eroglu et al., 2001). Therefore, e-commerce merchants need to present consumers with timely, sensory, and rich-visual information on product details (Petit et al., 2019), to reduce the uncertainty and perceived risk when making purchase decisions online.

Thanks to the rapidly developed interactive technology (e.g., virtual reality, augmented reality, and real-time live streaming), there are many product presentation formats for online consumption. Traditional e-commerce websites can use various visual presentations, such as static pictures, image zooming videos, product rotation, 3D product presentation, and virtual fitting rooms (Kim and Forsythe, 2008; Park et al., 2008; Algharabat et al., 2017; Petit et al., 2019). Owing to the spatial limitations of the Internet, the richness of media could increase the information transformation and communication effect (Daft and Lengel, 1986). Compared to verbal information in texts, pictures are seen as well-established predictors of consumers’ mental imagery (Wu et al., 2016). Yoo and Kim (2014) suggested that pictures are more effective than descriptions by texts, and pictures showing the method and scene of usage are more effective than pictures not showing them. Nowadays, online product presentation videos have increasingly become the popular way to display products online, because it has been proven to be more prosperous and vivid than pictures and texts with dynamic visual and auditory information (Jiang and Benbasat, 2007b; Vonkeman et al., 2017), it increases the perceived ease of imaging the product (Flaviaìn et al., 2017), and it provides the closest experience to the product in physical stores (Kumar and Tan, 2015).

In general, extensive research on online product presentation mainly focused on different kinds of product presentation formats. On the one hand, some studies especially compared product presentation text descriptions (Aljukhadar and Senecal, 2017), pictures (Wu et al., 2020; Jai et al., 2021), interactive images (Overmars and Poels, 2015), and virtual experience (Cowan et al., 2021) with videos. Some scholars think that product presentation video is better than other formats (Roggeveen et al., 2015); however, some scholars argued that product presented by pictures is more effective for search products (Huang et al., 2017). On the other hand, some research studies the combination of different kinds of product presentation. Jovic et al. (2012) discovered that the most effective combination format is text, picture, video, voice, and background music. Yue et al. (2017) recommended the combination of static photos, video, and 3D images.

Research has found that online product presentation formats significantly influence consumers’ positive attitudes and purchase intentions (Park et al., 2005; Jiang and Benbasat, 2007a; Verhagen et al., 2014; Visinescu et al., 2015). The online product presentation provides consumers with more product cues. It makes the products more vivid (Orús et al., 2017) and more accessible to evaluate (Jai et al., 2021). It also helps to increase consumer imagery fluency (Orús et al., 2017) and perception of interactivity (Kim and Forsythe, 2008) and decrease the perceived risk (Kim and Forsythe, 2008). In addition, it provides consumers with a sense of local presence (Algharabat et al., 2017) and psychological ownership and endowment (Brasel and Gips, 2014).

### Product presentation in live streaming and consumer’s purchase intention

As a new form of e-commerce, product demonstrations in live streaming have not received enough attention. Unlike traditional product video, live streaming provides a unique style of product presentation. The product presentation in live streaming is close to the actual using situations, showing products from various angles and providing trials by real people. The basic product information is introduced by anchors in words, rather than the traditional text product introduction on a webpage. This increases the amount of information transformation and the effectiveness of information understanding, which makes it easier for consumers to perceive the utilities of the product. In addition, anchors always try on products during the live streaming, such as eating food and trying clothes on and answer consumers’ questions interactively (Hilvert-Bruce et al., 2018).

Compared with the original display format, the most significant improvement of product presentation in live streaming is the rich, vivid, and interactive visual experience. On the one hand, product presentation in live streaming provides rich and tangible information. This experience is the primary sensory experience of shopping in the live room, and it increases the consumer’s perception of product quality and tangibility. For example, the static pictures, 360 spin rotation, and virtual mirror help consumers to form a clear mental representation of the product (Verhagen et al., 2016), get a sense of its physical characteristics, and even get an idea of how to use it (Schlosser, 2003; Jiang and Benbasat, 2007b). That is to say, the product presentation could help consumers to get cues about product functionality and its features (Coyle and Thorson, 2002). Therefore, the rich and tangible information presented in live streaming positively affects consumers’ perceived practical value of the product.

On the other hand, product presentation in live streaming provides vivid and interactive information. One fundamental problem with online shopping is that consumers lack sufficient awareness of products because they cannot check or try them. Jiang and Benbasat (2007b) found that diverse online product presentations provide more product cues, increase the perception of online products, and decrease information asymmetry. Studies discovered that high-quality pictures, three-dimensional (3D) images (Visinescu et al., 2015), and local presence (Verhagen et al., 2014) make online product presentations more vivid and interactive. In addition, a dynamic online product presentation, such as a product presentation video, could provide more specific clues to activate consumer mental imagery than a static online product presentation, such as pictures and texts (Overmars and Poels, 2015). In more depth, Huang et al. (2017) investigated how the interaction of static and dynamic displays of products and product types would affect consumer behavior. For experiential products (e.g., food or beauty), consumers would give higher evaluations if the product is displayed dynamically. Park et al. (2005) claimed that online apparel shopping is popular but also risky, because of the lack of sensory attributes displayed on the website, such as fabric hand, garment fit, color, and quality. Therefore, e-tailers need to create an attractive visual product presentation with some sense of fit and other tactile experiences (Szymanski and Hise, 2000). Three-dimensional (3D) product presentation enables consumers to visually inspect products by enlarging, zooming in or out on the product, and rotating the product (Algharabat et al., 2017). The interaction between anchors and consumers, especially the try-on behavior, makes the product presentation in live streaming more vivid and interactive and improves consumers’ purchase intention.

Generally speaking, product presentation in live streaming can help consumers better diagnose product quality, which enhances consumers’ shopping pleasure (Jiang and Benbasat, 2007a). Various formats of product presentations provide consumers virtual product experience (VPE) and enhance consumers to feel, touch, and even try products in a virtual online environment (Li et al., 2003). Based on these, this article proposed the first hypothesis:

H1: Product presentation in live streaming has a positive effect on consumer’s purchase intention.

### Mediating effect of consumer’s perceived product value

A consumer’s perceived product value is an overall mental evaluation of a particular good (Peterson and Yang, 2004). Product perceived value is about the assessment of consumers that they have received in terms of product quality and satisfaction and also that they have given in terms of money, time, and other costs. Research reveals that the perception of product value is a multidimensional and highly subjective evaluation of factors (Ruiz et al., 2008), including functional, symbolic, and experiential attributes (Boksberger and Melsen, 2011).

This article used the classic division of perceived product value dimensions: utilitarian and hedonic. On the one hand, utilitarian value is product-centric thinking, focusing on the functional, instrumental, and extrinsic cues of products (Hirschman and Holbrook, 1982). The two typical utilitarian values for consumers are monetary saving and convenience (Rintamäki et al., 2006). Monetary saving happens when consumers find discounted products, or when the prices are perceived as less than other stores. It reduces the consumer’s pain of paying (Chandon et al., 2000) and increases the consumer’s perceived utilitarian value of the product. Convenience is defined as a ratio of inputs (e.g., time and effort) to outputs (Holbrook, 1999). Seiders et al. (2000) pointed out that maximizing the speed and ease of shopping contributes to convenience. They defined four kinds of convenience, including access (reach a retailer), search (identify and select the essential products), possession (obtain desired products), and transaction (effect or amend transactions) convenience (Seiders et al., 2000).

On the other hand, the hedonic perception value of the product is self-oriented and self-purposeful (Holbrook, 1999). Generally speaking, consumers want entertainment and exploration during the consumption experience (Rintamäki et al., 2006). Studies found that themed environments, shows or events, and the overall store atmospherics could improve the entertainment of the shopping experience (Babin and Attaway, 2000). Hedonic value is sometimes a reaction to aesthetic features and is related to positive emotions evoked by the shopping experience. In addition, exploration is about the excitement of product or information search (Chandon et al., 2000). Consumers see shopping as an adventure, just enjoying browsing, seeking, and bargaining (Hausman, 2000).

Product presentation in the live-streaming context improves consumers’ perception of utilitarian product value from the convenient and monetary-saving parts. First, products in the living room are introduced and trailed by the anchors, and the linguistic and behavioral information output decreases time consumption. Once consumers enter the living room, they can easily access the products they are interested in, in the product lists or from the anchor’s display. They can also ask questions about the products to the anchors or the customer service staff. In addition, the design of the transaction process is easy and quick. These increase the perception of product utilitarian value, namely, access, search, possession, and transaction convenience. Second, product price seems cheaper than other sales channels. The anchors spend a lot of time discussing price discounts, such as receiving coupons, buy one get one, and other gifts. Therefore, consumers will count the price rationally and feel monetary savings.

At the same time, product presentation in the live-streaming context can also enhance consumer perceived hedonic product value from the entertainment and exploration aspects. First, the anchors in the live streaming are generally attractive, introduce products funnily, and make the consumers relaxed and delighted. Also, consumers could raise questions to anchor and interact with other audiences, which enhances their sense of immersion and offers a relatively real shopping scene (Liu et al., 2020). Finally, live-streaming selling is a new marketing strategy focused on consumers’ unnoticed interests, which leads consumers to explore new products. In reality, many consumers have no purchase needs at the beginning. Still, after watching the introduction in the living room, they become interested in the product and intend to buy it.

A consumer’s perceived product value is one of the most critical determinants of a consumer’s purchase intention (Chang and Wang, 2011). When consumers shop online, the utilitarian value of the website could positively affect their flow experience and then affect their intention of continuing to consume (Chang and Chen, 2014). As for the hedonic shopping value, it will affect consumers’ information search propensity and purchase intention (Wang, 2010). In addition, hedonic values have a direct impact on consumers’ perceived uniqueness, leading to place dependence, frequent visits, and longer shopping time (Allard et al., 2009).

Therefore, product presentations in live streaming enhance the two kinds of perceived product value, by providing external information and generating self-cognitions for consumers. After obtaining product-related information, consumers would psychologically reflect on the meanings and value of the information in the product. Thus, the higher the value of information, the higher the consumer’s perceived product value (Zhang and Merunka, 2015). At the same time, the higher the value of information indicates that consumers have an in-depth and comprehensive understanding of the product and thus feel the product is sincere and reliable (Manfred et al., 2012). Hence, this article used perceived product value as mediating variable and proposed the second hypothesis:

H2: Consumer’s perceived product value mediated the positive effect of the product presentation and consumer’s purchase intention.

### Moderating effect of time pressure on consumption

Time pressure is an anxious emotional response that arises from the decision-maker’s lack of time to complete tasks within a specific deadline (Svenson and Edland, 1987). Time pressure could be divided into subjective time pressure and objective time pressure. The subjective time pressure is mainly determined by the discount rates, while the objective time pressure is determined by the promotion time constraints. Discount rates and time constraints constitute opportunity costs, lead to consumers’ perceived time pressure, and then affect consumers’ decisions (Zhu and Zhang, 2021).

Time pressure has a moderating effect on the mediation relationship between product presentation and consumer purchase intention. Time pressure reduces consumers’ information search during the purchase decision process (Beatty and Smith, 1987). Under time pressure, consumers spend significantly less time searching for information, especially unbiased information sources (Murray, 1983). In addition, their cognitive closure is more inclined to intuitive heuristics (Murray, 1983), relying on experience or intuition to make decisions. Under this condition, consumers tend to exaggerate the perceived benefits, ignore possible risks, look for evidence to support their ideas, and pay less or no attention to evidence that denies their views. They have less time to attain and analyze other rich information rather than that got from product presentation, and they make purchase decisions impulsively and fast. That is to say, for consumers with high time pressure, their purchase intention primarily relied on information obtained from product presentations. Therefore, the limited time constraint, or time pressure, may enhance the positive effect of product presentation on consumers’ purchase intention. Based on these, the third hypothesis was proposed:

H3: Time pressure moderates the effect of product presentation on consumers’ purchase intention. For consumers under a high level of time pressure, product presentation is positively associated with consumer purchase intention; for consumers under a low level of time pressure, the positive impact of product presentation on purchase intention is attenuated.

**FIGURE 1:**
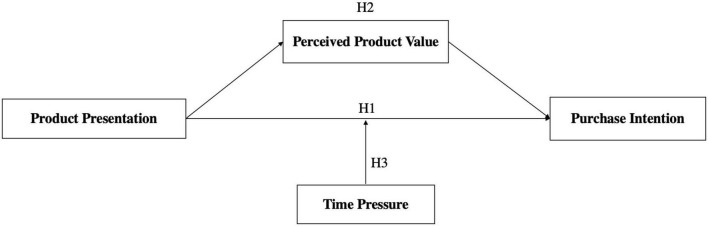
The research framework.

Based on the above hypotheses, the research framework is shown in Figure 1.

## Study 1

Study 1 was a self-reported survey, to explore the main effect of product presentation on consumers’ purchase intention and the mediating effect of the perceived product value. In Study 1, participants were asked to recall their consumption experience in live streaming and answer related questionnaires.

### Participants

The questionnaire was designed and released through the Credamo platform. We received 249 answers. There were 198 qualified responses eventually, after excluding questionnaires that showed too long/short duration, regular answering patterns, incomplete information, and failed to pass screening questions. Among them, 76 (38.4%) were male participants, 141 (71.2%) were 21–30 years old, and 51 (25.8%) were 31–40 years old. More demographic information was shown in Table 1.

**TABLE 1:** Description of participants’ demographics in Study 1.

Age	*N*	Percentage (%)
0–20	3	1.5
21–30	141	71.2
31–40	51	25.8
41–50	3	1.5
**Education**		
High school	2	1.0
Associate’s degree	17	8.6
Bachelor’s degree	163	82.3
Master’s degree	14	7.1
Ph.D. degree	2	1.0
**Occupation**		
Student	17	8.6
State-owned enterprises	46	23.2
Public institutions	33	16.7
Civil servant	2	1.0
Private enterprises	89	44.9
Foreign-invested enterprises	11	5.6
**Monthly income (RMB)**		
<3,000	13	6.6
3,000–4,999	32	16.2
5,000–7,999	81	40.9
8,000–10,000	46	23.2
≥10,000	26	13.1
**Monthly consumption (RMB)**		
<1,000	19	9.6
1,000–1,999	85	42.9
2,000–2,999	65	32.8
3,000–4,000	25	12.6
≥4,000	4	2.0

*N* = 198.

### Procedures and measures

After obtaining informed consent, participants were asked to recall their last live-streaming watching and shopping experience and answer related questions. First, the detailed information was based on the consumption experience. They were asked whether they watched the consumption live streaming and whether they bought products in the live-streaming room. They were also required to write down this consumption experience with detailed information, such as the brand, product category (e.g., clothing, food, and cosmetics), and price, to enhance the recalling effect. Second, product presentation was measured with mature scales (α = 0.62; Farrelly et al., 2019). Third, product purchase intention was measured with a 4-item scale adapted from mature scales (α = 0.83; Huang et al., 2013). Fourth, perceived product value was measured with 12 items in total (α = 0.88; Mathwick et al., 2001; Loiacono et al., 2007) for the utilitarian and hedonic value. All the items were measured with a 7-Likert scale, with 1 = strongly disagree and 7 = strongly agree. Finally, demographics were collected, including gender, age, occupation, highest education, monthly income, and monthly consumption.

## Results

### Common method bias check

Given the nature of the single-shot cross-sectional survey, we first checked whether there was a common method bias before the formal data analysis. Harman’s one-factor analysis was conducted (Podsakoff and Organ, 1986), by including all of the items of critical variables for an exploratory factor analysis using a maximum likelihood solution. The results showed that four factors emerged with eigenvalues larger than 1.00, indicating that more than one factor underlies the data. In addition, the first factor accounted for only 39.12% of the total variance, suggesting that the common method variance may not be a severe concern in the present study (Eby and Dobbins, 1997).

### The main effect of product presentation on consumer’s purchase intention

Descriptive statistics and correlation coefficients of key variables are presented in Table 2. To test the main effect of product presentation on product purchase intention in the live streaming, regression analysis was conducted by two models (refer to Table 3). In Model 1, we regressed the product presentation on consumers’ purchase intention. Model 2 revealed that after controlling for demographic variables such as gender, age, education, occupation, monthly income, and monthly expenditure, product presentation also positively predicted customers’ purchase intention (β = 0.71, *t* = 13.75, *p* < 0.000, refer to Table 3) and, thus, H1 was supported.

**TABLE 2:** Description and correlation of variables in Study 1.

	Mean	SD	Product perceived value	Purchase intention
Product presentation	5.85	0.58		
Perceived product value	5.80	0.54	0.76**	
Purchase intention	5.81	0.57	0.72**	0.79**

**p < 0.01.

**TABLE 3:** Regression analysis for Study 1.

Purchase intention	Model 1		Model 2	
	β	*t*	β	*t*
Product presentation	0.72**	14.43	0.71**	13.75
Gender			−0.11*	−2.05
Age			−0.12*	−2.28
Occupation			0.07	1.33
Education			0.12*	2.37
Monthly income			−0.003	−0.05
Monthly consumption			0.02	0.25
*R* ^2^	0.52		0.55	
Adjusted *R*^2^	0.512		0.533	
*F*	208.09		33.12	

*N* = 198; *p < 0.05, **p < 0.01.

### Mediation effect analysis

We predicted that the perceived product value would mediate the effect of product presentation on product purchase intention. A 5,000 resampling bootstrapping mediation analysis using product presentation as the predictor, perceived product value as the mediator, and product purchase intention as the dependent variable (Hayes, 2018, Model 4) confirmed this prediction. The analysis revealed a significant omnibus index of mediation (Effect = 0.43, SE = 0.07, 95% CI: [0.30, 0.58]). Thus, H2 was supported.

## Study 2

Study 2 was a survey-based behavioral experiment. The aim of Study 2 was to test the main effect of product presentation on consumer purchase intention, and the mediation effect of perceived product value, namely to verify H1 and H2.

### Participants

This experiment was designed and distributed through the online survey platform Credamo.^1^ A total of 83 subjects, who had not joined Study 1, participated in the formal experiment, and 23 subjects were excluded because of too long or too short response time, inconsistent responses, and wrong answers for the attention check. Among the final 60 participants, 29 (48.3%) were male participants, and the average age was 29.08 years (SD = 5.54, Min = 18, Max = 42). More demographics are shown in Table 4.

**TABLE 4:** Description of participants’ demographics in Study 2.

Education	*N*	Percentage (%)
High school	1	1.7
Associate’s degree	4	6.7
Bachelor’s degree	42	70.0
Master’s degree	13	21.7
Ph.D. degree	0	0
**Occupation**		
Student	13	21.7
State-owned enterprises	12	20.0
Public institutions	4	6.7
Civil servant	3	5.0
Private enterprises	24	40.0
Foreign-invested enterprises	4	6.7
**Monthly income (RMB)**		
<3,000	10	16.7
3,000–4,999	14	23.3
5,000–7,999	18	30.0
8,000–10,000	8	13.3
≥10,000	10	16.6
**Monthly consumption (RMB)**		
<1,000	21	35.0
1,000–1,999	25	41.7
2,000–2,999	11	18.3
3,000–4,000	2	3.3
≥4,000	1	1.7

*N* = 60.

### Procedures and measures

Study 2 was a one-factor (product presentation: high vs. low) between-subject design. The final 60 subjects were randomly assigned to one of two experimental groups, with 30 people in each condition. Before the formal experiment, participants signed informed consent online. They were guaranteed anonymity and allowed to discontinue the experiment at any time. They were told that this was a sociological study that consisted of several unrelated sub-surveys. After the answer was qualified and accepted, each participant would be paid 5 yuan in renminbi (RMB).

Participants were first shown the same live-streaming clip for approximately 20 s. It was cut from the “Ear Gourmet” living room and presented the product of chocolate. This video introduced the basic product information, including the chocolate brand, original country, price, and four kinds of flavors.

Second, the different conditions were primed with different descriptions of the product presentation information. The high level of product presentation is primed by enough information about this chocolate in detail, such as the origin, raw materials, functional groups, product positioning, and applicable scenarios. In addition, the participants were told that the anchor also introduced the information about this chocolate in detail through various behaviors in the live streaming, including the anchor’s tasting, the assistant’s tasting, product detail display, and interactive Q&A. However, in the group of low-level product presentation, participants were told that the anchor did not introduce other product information, except for the above information got in the video. Furthermore, the anchor did not show the product through behaviors in the live streaming, such as the anchor’s tasting, the assistant’s tasting, product detail display, and interactive Q&A.

Third, participants were asked to recall video contents and then answer their purchase intention with four items (α = 0.93; Huang et al., 2013). Fourth, manipulation checks and attention checks were tested. The questions for the manipulation check used the scales of product presentation (α = 0.91; Farrelly et al., 2019) with seven items, such as “Anchor introduced objective attributes of products, such as ingredients and specifications” and “There are product trials sessions in live streaming.” Furthermore, there are two questions for the attention check, about the contents of the video or text reminder. Fifth, product perceived value was measured with six items for the utilitarian value (α = 0.91; Loiacono et al., 2007) and six items for the hedonic value (α = 0.93; Mathwick et al., 2001). The example items are “The products recommended in live streaming meet my functional demands for such products” and “I think the live streaming entertains me.” Finally, the demographics were collected, including gender, age, highest education, work, monthly income, and monthly consumption.

## Results

### Manipulation check of product presentation

The results indicated that there is a significant difference in the perception of product presentation between the high-level (Mean = 5.73, SD = 0.90) and the low-level groups (Mean = 2.80, SD = 0.59), *t* = −15.01, *p* < 0.000. Therefore, the manipulation of high and low levels of product presentation succeeded.

### The main effect of product presentation on consumer’s purchase intention

The independent sample *t*-test revealed that product presentation had a positive main effect on consumers’ purchase intention. Participants in the high product presentation had higher purchase intention (M_high product presentation_ = 5.70, SD_high product presentation_ = 0.99) than those in the low group (M_low product presentation_ = 3.92, SD_low product presentation_ = 1.40), *t* = −5.71, *p* < 0.000. Thus, H1 was supported.

### Mediation effect analysis

We predicted that consumers’ perceived product value would mediate the effect of product presentation on product purchase intention. A 5,000 resampling bootstrapping mediation analysis confirmed this prediction, using product presentation as the predictor, perceived product value as the mediator, product purchase intention as the dependent variable (Hayes, 2018, Model 4), and demographics as control variables. The analysis revealed a significant omnibus index of mediation for product presentation (Effect = 0.99, SE = 0.26, 95% CI: [0.54, 1.56]). Thus, H2 was supported.

## Study 3

Study 3 was a survey-based behavioral experiment to explore further the mediating effect of product value perception and moderated mediation effect of time pressure in the clothing consumption scenario.

### Participants

A total of 176 participants were recruited from the sample database on Credamo. After excluding 58 answers that were too long or too short response time, inconsistent responses, and wrong answers for the attention check, 118 valid answers were reserved. Among them, 52 (44.1%) were male participants, and the average age was 28.53 years (SD_age_ = 6.72, Min_age_ = 19, Max_age_ = 52). More demographic information is shown in Table 5.

**TABLE 5:** Description of participants’ demographics in Study 3.

Education	*N*	Percentage (%)
High school	2	1.6
Associate’s degree	15	12.7
Bachelor’s degree	87	73.7
Master’s degree	14	11.9
Ph.D. degree	0	0
**Occupation**		
Student	32	27.1
State-owned enterprises	24	20.3
Public institutions	5	4.2
Civil servant	2	1.7
Private enterprises	50	42.4
Foreign-invested enterprises	5	4.2
**Monthly income (RMB)**		
<3,000	30	25.4
3,000–4,999	20	16.9
5,000–7,999	29	24.6
8,000–10,000	17	14.4
≥10,000	22	18.6
**Monthly consumption (RMB)**		
<1,000	52	44.1
1,000–1,999	44	37.3
2,000–2,999	14	11.9
3,000–4,000	7	5.9
≥4,000	1	0.8

*N* = 118.

### Procedures and measures

Study 3 followed a 2 (product presentation: high vs. low) * 2 (time pressure: high vs. low) between-subject design. Participants were recruited to join in a survey on product evaluation in live streaming. They signed informed consent online, guaranteed anonymity, and were allowed to discontinue the experiment at any time. After the answer was checked and accepted, each participant would be paid 5 yuan in RMB.

First, watch the same video of the product. All participants were asked to watch a short video carefully, which was an excerpted video from Anta’s live-streaming room. The anchor introduced the black and white panda sneakers, the same style for men and women. The price of this product in the live-streaming room is 229 yuan in RMB. Second is the manipulation of different levels of product presentation. Participants were randomly assigned to read different descriptions of the information in the live room, to prime consumers’ different perceptions of the product presentation and time pressure. The product presentation was primed with detailed/brief descriptions of the shoes, to manipulate the high/low level of product presentation. The high/low time pressure was primed by “The low price and coupons in the live-streaming room are valid for a short/long time, leaving a short/long time for consumers to make purchasing decisions.” “The anchor continues to/does not continue to urge consumers to quickly buy” (Benson and Svenson, 1993). Third, participants completed the purchase intention scale (α = 0.93) and product value perception scale (α = 0.96; Mathwick et al., 2001; Loiacono et al., 2007). Then, participants indicated their agreement on two scales as a manipulation check for product presentation (α = 0.89; Farrelly et al., 2019) and time pressure (α = 0.95; Svenson, 1992). All measurements were based on a 7-point Likert scale (1 = Strongly disagree; 7 = Strongly agree). Finally, participants reported their demographics as identical to that in Study 2. We also included an attention check in the middle of the process.

## Results

### Manipulation check of the product presentation and time pressure

As we expected, participants in the high product presentation perceived high product presentation information (M_high product presentation_ = 5.68, SD_high product presentation_ = 0.87) more than those in the low group (M_low product presentation_ = 3.69, SD_low product presentation_ = 1.08), *t* = −10.99, *p* < 0.000. Moreover, the *t*-test revealed that there is also a significant difference in the perception of time pressure between high and low groups, *t* = −15.81, *p* < 0.000, M_high time pressure_ = 5.73, SD_high time pressure_ = 1.08, M_low time pressure_ = 2.28, SD_low time pressure_ = 1.27. The result showed that the manipulation of the product presentation and time pressure was effective.

### The main effect of product presentation on consumer’s purchase intention

The independent sample *t*-test revealed that product presentation had a positive main effect on consumers’ purchase intention. Participants in the high product presentation had higher purchase intention (M_high product presentation_ = 5.80, SD _high product presentation_ = 0.72) than those in the low group (M_low product presentation_ = 4.06, SD_low product presentation_ = 1.55), *t* = −5.71, *p* < 0.000. Thus, H1 was supported.

### Mediation effect of perceived product value

Based on Model 4 in PROCESS (Hayes, 2018), we conducted a 5,000 resampling bootstrapping mediation analysis, using product presentation (0 = low level, 1 = high level) as the predictor, consumer perceived product value as the mediator, and consumer’s purchase intention as the dependent variable. The results confirmed a significant mediation effect of product value (Effect = 1.15, SE = 0.18, 95% CI: [0.80, 1.50]). Therefore, H2 was supported again.

### Moderation effect

Following Model 5 of the PROCESS Macro (Hayes, 2012), we performed a 5,000 resampling bootstrapping moderated mediation analysis with product presentation (0 = low level, 1 = high level) as the independent variable, perceived product value as the mediator, time pressure (0 = low level, 1 = high level) as the moderator, and consumer’s purchase intention as the dependent variable. The results indicated a moderated effect of time pressure perception (Effect = 0.70, SE = 0.26, 95% CI: [0.20, 1.21]). In particular, for consumers with a low level of time pressure, the main effect of product presentation on purchase intention was not significant (Effect = 0.27, SE = 0.19, 95% CI: [−0.10, 0.64]); However, when the time pressure perception was high, the positive effect of product presentation on consumer’s purchase intention was significant (Effect = 0.97, SE = 0.20, 95% CI: [0.57, 1.38]). In addition, the mediation effect of “product presentation→product value perception→purchase intention” was significantly positive (Effect = 1.11, SE = 0.18, 95% CI: [0.78, 1.46]). Therefore, H3 was supported.

## Conclusion and implications

### Conclusion

This article focused on the formats of product presentation in the context of live streaming. It investigated the relationship between product presentation and consumer purchase intention and the specific psychological mechanisms. Based on three studies, this article found that product presentation in live streaming had a positive effect on consumers’ purchase intention. Also, it tested the mediating effect of consumer perceived product value, both utilitarian and hedonic values, and the moderated mediation effect of time pressure. The results indicated that product presentation, especially the high level of vivid, rich, and interactive information displayed in the live room, increased consumers’ perception of product value, thereby improving consumers’ purchase decisions. However, the boundary of the above effect is the time pressure perception. When consumers considered that the time pressure is high, they had less time to access, process, and analyze related product information, and the positive effect of product presentation on purchase intention was enhanced.

### Theoretical contributions

Theoretically speaking, this article extended the literature on product presentation, by providing a new research context of live streaming. Previous research mainly focused on product display in e-commerce on the webpage, involving text, pictures, videos, and other dynamic display methods (Overmars and Poels, 2015; Wu et al., 2020; Cowan et al., 2021; Jai et al., 2021). To some extent, this is a kind of two-dimensional (2D) and sometimes three-dimensional (3D) product presentation, which is a one-way information input from the webpage to consumers. However, the product presentation in the live-streaming scene is a real-time, two-way, and 3D combination display (Sjöblom and Hamari, 2017). Products are presented by oral introductions, tryouts in action, and answers to consumers’ personalized questions by the anchor. The Q&As between anchors and consumers realize two-way information transmission, which helps consumers learn more rich, interactive, and tangible product information. In addition to the differences in the specific forms of product displays, the particular mechanism of the product presentation on consumers’ decisions also needs to be re-examined. Prior studies have found that the product display on the webpage works through perceived risk (Jiang and Benbasat, 2007b), mental imagery (Overmars and Poels, 2015), vividness (Orús et al., 2017), interactivity (Kim and Forsythe, 2008), local presence (Algharabat et al., 2017), and so on. However, this article found that rich product presentation helps consumers understand the utilitarian and hedonic value of the product in an all-around way, thereby promoting their purchase intention.

In addition, this article extended the literature on live streaming and consumer behaviors, by providing a new research perspective on product presentation. Live streaming is a rapidly emerging Internet-age phenomenon. Scholars currently studied the characteristics of the anchor and the consumers, the characteristics of the anchor (anchor type; Huang et al., 2021), the fit between the anchor and products (Park and Lin, 2020), typology of seller’s sales approach (Wongkitrungrueng et al., 2020), consumer’s social motivation to watch live streaming (Hilvert-Bruce et al., 2018), personal characteristics for live-streaming addiction (state boredom; Zhang and Li, 2022), and so on. However, less research cares about product presentation currently. It seems that the particularity of the live streaming is the anchor. But in fact, this real-time video greatly enriches the form and content of product display. Therefore, this article studied the effect of product presentation on consumers’ purchase decisions. At the same time, this article also considered time pressure, a new factor in the living room, as a moderator. Limiting time is often used in the live room, but whether the substantial effect is good or bad is not conclusive. Therefore, simultaneous consideration of product presentation and time pressure makes a theoretical contribution to the study of live marketing.

### Managerial implications

From the marketing practices, the results of this article would support brands, anchors, and consumers. On the one hand, the formats of product presentation in the living room should be well designed. This article concluded that a high level of product presentation has a positive effect on consumer purchase intention. Therefore, brands and anchors could get enlightenment on how to fully use different presentation methods to maximize consumers’ perceived value and purchase intention. Within the live-streaming shopping, anchors are supposed to focus on introducing the characteristics of products, optimizing the performance of trials and Q&As, and using product value and stories as supplements. By reasonably assigning the significance of a high level of presentation information, consumers could perceive utilitarian and hedonic product value faster and better. This display enhances consumers’ shopping pleasure (Jiang and Benbasat, 2007a) and provides a similar experience to shopping in physical stores (Kumar and Tan, 2015). Hence, consumers could be delighted, would like to purchase products, and stay in the live room for a long time (Jovic et al., 2012).

On the other hand, anchors should enhance the role of time pressure in a timely manner to achieve the effect of stimulating consumer purchase. In practice, the time constraints for each product in the live room are very strict. It seems that the less time left to the consumer, the more likely the consumer is to buy impulsively. However, how to control the purchase time embodies the art of management. Excessive time constraints can degrade the shopping experience for consumers. Therefore, if brands or anchors hope to stimulate consumers’ impulse buying by limiting the purchase time, they should highlight the product valve (e.g., benefits and scarcity of the product) as much as possible and improve the transaction utility of the products (Zhu and Zhang, 2021).

### Limitations and future research

There are two deficiencies in this article, and future research can make up for two aspects. First is the division of product presentations. In this article, product presentation is considered as a whole, and the differential effects of its high and low levels on consumer decisions were studied. In the future, researchers could divide product presentation as intrinsic cues (e.g., flavor and aroma cues for beer) and extrinsic cues (e.g., price, store image; Olson and Jacoby, 1972). Second is the abundance of stimuli materials. In this article, the stimuli chosen from the live streaming are food and appeal, which are the top two popular categories sold for live streaming. In the future, more kinds of products (such as terroir products or tourism products) could be studied in order to see whether the results are still robust.

## Data availability statement

The raw data supporting the conclusions of this article will be made available by the author, without undue reservation.

## Ethics statement

The studies involving human participants were reviewed and approved by the School of Economics and Management, Beijing Jiaotong University. The patients/participants provided their written informed consent online to participate in this study.

## Author contributions

NZ: conceptualization, methodology, writing and editing, funding acquisition, and approved the submitted version.

## Footnotes

1
https://www.credamo.com/#/
